# Factors related to the social network of core members of elderly care service social organizations: a cross-sectional study

**DOI:** 10.1186/s12913-022-08545-7

**Published:** 2022-09-10

**Authors:** Zhengsheng Wang, Xingxi Zhang, Liu Liu, Ling Tang, Ying Zhu, Zhongliang Bai, Ren Chen

**Affiliations:** 1 School of Health Services Management, Anhui Medical University, Hefei, 230032 China; 2 The Second Affiliated Hospital of Anhui Medical University, Hefei, 230601 China; 3 The First Affiliated Hospital of Anhui University of Chinese Medicine, Hefei, 230031 China; 4 Suzhou Hospital Affiliated to Anhui Medical University, Suzhou, 234000 Anhui China

**Keywords:** Aging, Social network, Elderly care services, Social organizations, Core members

## Abstract

**Background:**

The social network of the core members of elderly care service social organizations could affect the performance of the organization, while studies concerning its related factors are limited. We aimed to explore factors that are associated with the social network of core members from elderly care service social organizations and provide references and suggestions for improving elderly care services.

**Methods:**

This cross-sectional study employed a multi-stage stratified sampling method, and collected data concerning social network, demographic information and occupation. Univariate analysis and binary logistic regression were used to analyze factors that could affect the social network of the core members.

**Results:**

Our results demonstrated that there is low social network of core members of elderly care social organizations. Out of the total membership, men (AOR = 1.708; 95%CI: 1.034–2.823), those with senior high school education (AOR = 1.923; 95%CI: 1.053–3.511), those with a college degree and above (AOR = 3.010; 95%CI: 1.591–5.692) and those that receive awards related to elderly care services (AOR = 2.260; 95%CI: 1.285–3.976) were associated with higher social network scores.

**Conclusions:**

Our data successfully characterized the social status of core members of elderly care organizations. Therefore, health care professionals and policy makers in social organizations should use this knowledge in the care and service provision to the elderly; and implement actions that would promote networking in social organizations.

**Supplementary Information:**

The online version contains supplementary material available at 10.1186/s12913-022-08545-7.

## Background

Rapidly growing aging population has posed major challenges in the world [1]. It is estimated that by 2050, people aged 65 years and above will account for 27.9% of the total population in China [2]. China is moving into an aging society and thus there is tremendous pressure on elderly care services. Encouraging social organizations to provide care services for the elderly is a feasible strategy for curbing these challenges. previous studies have demonstrated that social organizations play an important role in these services [3–5]. Besides, introduction of social organization services in health care is of great importance in disease prevention as well as the realization of universal basic public healthcare [6, 7]. Therefore, empowering the development of social organizations in the care for the elderly should be a priority.

Other studies have shown that cross-sector collaboration, which refers to the cooperation of government, social organization, and other institutions [8], could improve the quality and efficiency of social services, especially in health care services for the elderly [9]. The studies concluded that social network, which reflects the connection and interaction between different stakeholders, such as friendships, relatives, neighborhood, and social participation [10], could facilitate cross-sector collaboration [11–13]. Core members in social organizations include founders, leaders, legal representatives as well as managers who are familiar with the organizations. In addition, studies have demonstrated that social network of organizational leaders does not only affect employees’ performance but the employees’ job satisfaction and willingness to leave [14, 15]. Another study showed that the social network of organizational leaders could affect the performance of the organization, thus affecting the sustainable development of organizations [16]. Therefore, there is a need to define factors related to the social network of core members in elderly care social organizations. Here, we performed a cross-sectional study to explore factors related to the social network of core members of elderly care services social organizations in Anhui Province, China.

## Materials and methods

### Study design and data collection

This study was conducted in November and December 2019, in Anhui Province, China, and was approved by the Biomedical Ethics Committee of Anhui Medical University (No. 20180181).

We employed a multi-stage stratified sampling method to collect samples used in this study. According to the 2016 statistics of Anhui Statistics Bureau and related literature research, the economic level of Anhui Province is high in the middle, medium in the south, and low in the north [17, 18]. In order to make the study area more representative, we selected Lu'an and Huainan in central Anhui, Anqing, and Chizhou in southern Anhui, and Fuyang and Suzhou in northern Anhui, based on geographical location and economic development level. All the districts of these cities (15 districts) were included in the survey. We then randomly selected half of the social organizations in the elderly services in each district as survey organizations. If the number of core members in an organization is less than or equal to 3, all core members in the organization will be included in the survey. If the number of core members in an organization is greater than 3, then 3 of them will be randomly selected for the survey. The core members included organization founders, organization leaders, legal representatives, and organization managers. More details about our sampling process had been described previously [19].

The investigators were trained and experienced graduate students from Anhui Medical University, and the investigation was coordinated by the civil affairs departments of each district. The investigators were divided into 4 teams, headed by workers from the civil affairs department, and conducted a face-to-face interview questionnaire survey on the subjects. Prior to the survey on the subjects, the investigators explained the contents of the questionnaire, assured the confidentiality of the subject's personal information and privacy, as well as obtained informed consent. In this study, a total of 315 core members of social organizations in the elderly care services participated in the questionnaire survey, seven core members were excluded due to missing values. Finally, 308 of 315 (97.8%) core members were included in the data analysis.

### Measurement of social network

Based on the World Bank’s Social Capital Assessment Tool coupled with previous studies [20–23], we developed an individual social network measurement questionnaire. Then, through many consultations with authoritative experts in the professional field and pre-investigation, we modified and improved the questionnaire to ensure its scientificity and feasibility of the questionnaire. The questionnaire's Cronbach's α was 0.885, indicating that our questionnaire has good internal consistency. The questionnaire consisted of two parts; social network with government departments and social network with social institutions, with a total of 7 items. The social network with government departments assessed the number of acquaintances of core members in the Civil Affairs Department, the number of acquaintances in the Health Committee, the number of acquaintances in the Medical Security Bureau, and the number of acquaintances in other government departments. On the other hand, the social network with social institutions measured the number of acquaintances of the core members in the community committee, the number of acquaintances in the social organization federation as well as the number of acquaintances in other elderly care service social organizations. For each item of the social network, we used Likert's five-point scale to measure the number of acquaintances of the core members (1 = “0”, 2 = “1–5”, 3 = “6–10”, 4 = “11–15”, and 5 = “16 and above”). The total score of the social network of each survey object was obtained by summing up the scores of the 7 items, ranging from 7–35 points. Using the median score, we divided the social network scores into high and low categories [20]. The detailed questionnaire can be found in the Supplementary file 1.

### Measurement of other variables

Information on the demographic variables of the core members included gender (male, female), age (≤ 40,41–49, ≥ 50 years), nationality (Han, others), education (junior high school and below, senior high school, college degree and above) as well as marital status (married, others). We also collected occupational information such as professional title (have, not have), full-time staff (yes, no), organization's length of service (≤ 1 year, 2–5 years, ≥ 6 years), length of service in the elderly care field (≤ 1 year, 2–5 years, ≥ 6 years), obtained practice certificate (yes, no), obtained professional qualification certificate (yes, no), received training related to elderly care services (yes, no), reported by the media for elderly care services (yes, no) as well as whether received awards related to elderly care services (yes, no).

### Statistical analysis

We first tested the normality of the scores of the social network. We used the median (interquartile range) to present the data since it was non-normal. We then took the social network scores as a dependent variable, while demographic characteristics and occupational information were taken as independent variables, and were used to perform univariate analysis of the potential influencing factors of core members’ social network. Non-parametric test was used to examine the differences in social network scores of the core members in different groups, and the variables were expressed in the form of numbers and percentages. Mann–Whitney test was used for the examination of variables with only two groups, while variables with three or more groups were evaluated using the Kruskal–Wallis test.

We used individual social network scores as dependent variables (1 = high, 0 = low), and then statistically significant variables in the non-parametric test were included in the binary logistic regression model and used for analysis. The analysis adjusted for the potential covariates such as gender, education, organization's length of service, reported by the media for elderly care services, and received awards related to elderly care services. Results of the binary regression logistic analysis were expressed in adjusted odds ratio (AOR), with an associated 95% confidence interval (95% CI).

All the statistical analyses were performed in IBM SPSS Statistics 23.0 software, and a *P*-value < 0.05 was taken as statistically significant.

## Results

### Descriptive Analysis and Univariate Analysis

We characterized the social demographics of core members and performed the univariate analysis of the social network as shown in Table 1. Out of the total respondents, 178 were men (57.8%) and 130 were female (42.2%). 174 respondents were over 50 years old (56.5%), and Han nationality accounted for 99%. Further analysis showed that 105 respondents had a college degree or above (34.1%), while only 62 respondents had professional titles (20.1%). 289 respondents were full-time staff (93.8%), and 284 respondents were married (92.2%). 119 respondents had worked in the same organization for 2–5 years (38.6%), while 122 respondents had worked in the elderly care services for more than 6 years (39.6%). 260 respondents lacked a practicing certificate (84.4%), but 203 respondents had obtained a professional qualification certificate (65.9%). In terms of training, 210 respondents had training related to elderly care services (68.2%). 174 respondents had been reported by the media because of the elderly care services they were engaged in (56.5%), while 84 respondents had received awards related to elderly care services (27.3%).

**Table 1:** Social demographic characteristics of core members and the results of the univariate analysis of social networks in social organizations (*N* = 308)

Variables	N (%)	Social networks scores(M(IQR))	Z/χ^2^	*P*-value
**Gender**			-2.341	0.019*
Male	178(57.8)	14(8)		
Female	130(42.2)	12(8.25)		
**Age(years)**			3.068	0.216
≤ 40	74(24)	12(8)		
41–49	60(19.5)	14(9.75)		
≥ 50	174(56.5)	14(8)		
**Nationality**			-0.323	0.747
Han	305(99)	14(8)		
Others	3(1)	15(8)		
**Education**			25.904	< 0.001*
Junior high school and below	91(29.5)	11(7)		
Senior high school	112(36.4)	14(8)		
College degree and above	105(34.1)	16(10)		
**Marital status**			-0.330	0.741
Married	284(92.2)	14(8)		
Others	24(7.8)	11.5(9)		
**Professional title**			-0.217	0.828
Have	62(20.1)	14(8.5)		
Not have	246(79.9)	14(8.25)		
**Full-time staff**			-1.015	0.310
Yes	289(93.8)	14(8)		
No	19(6.2)	14(9)		
**Organization's length of service(years)**			7.627	0.022*
≤ 1	98(31.8)	14(8)		
2–5	119(38.6)	12(8)		
≥ 6	91(29.5)	14(9)		
**Length of service in the elderly care field(years)**			4.906	0.086
≤ 1	77(25)	14(8.5)		
2–5	109(35.4)	13(7)		
≥ 6	122(39.6)	14(9.25)		
**Obtained practice certificate**			-0.246	0.806
Yes	48(15.6)	14(10)		
No	260(84.4)	14(8)		
**Obtained professional qualification certificate**			-1.391	0.164
Yes	203(65.9)	14(7)		
No	105(34.1)	12(9)		
**Received training related to elderly care services**			-1.796	0.072
Yes	210(68.2)	14(8)		
No	98(31.8)	12(7)		
**Reported by the media for elderly care services**			-4.788	< 0.001*
Yes	174(56.5)	17(10.75)		
No	134(43.5)	12(7)		
**Received awards related to elderly care services**			-3.934	< 0.001*
Yes	84(27.3)	15(9)		
No	224(72.7)	12(8)		

^*^Represents statistically significant

On the other hand, results of social network scores of core members of the elderly care service social organizations showed that the lowest score was 7 points, while the highest score was 35 points, and the median score was 14 points. The univariate analysis results showed that among all the variables, gender, education level, organization's length of service, being reported by the media for elderly care services, and awards related to elderly care services had a significant impact on the social networks.

### Results of the binary logistic regression analysis

Data from the binary logistic regression analysis of the relationship between social network scores and the basic personal situation of core members were as shown in Table 2. The Hosmer–Lemeshow test showed that there was no significant difference between the predicted value and the true value, and the model fits well (*P* = 0.808). Besides, social network scores were related to gender, education level, and whether one received awards related to elderly care services. Compared with the reference groups, men (AOR = 1.708; 95%CI: 1.034–2.823), people with senior high school education (AOR = 1.923; 95%CI: 1.053–3.511), people with a college degree and above (AOR = 3.010; 95%CI: 1.591–5.692), as well as those who received awards related to elderly care services (AOR = 2.260; 95%CI: 1.285–3.976) were associated with higher social network scores.

**Table 2:** Binary logistic regression analysis of the social network scores

Variables	B	SE	Wald	*P*-value	AOR (95%CI)
**Gender**					
Female(REF.)					
Male	0.536	0.260	2.518	0.037	1.708(1.034–2.823)
**Education**					
Junior high school and below(REF.)					
Senior high school	0.654	0.307	4.534	0.033	1.923(1.053–3.511)
College degree and above	1.102	0.325	11.481	0.001	3.010(1.591–5.692)
**Organization's length of service(years)**					
≤ 1(REF.)					
2–5	-0.249	0.300	0.693	0.405	0.799(0.433–1.402)
≥ 6	0.224	0.328	0.465	0.495	1.251(0.658–2.378)
**Reported by the media for elderly care services**					
No(REF.)					
Yes	-0.413	0.260	2.518	0.113	0.662(0.397–1.102)
**Received awards related to elderly care services**					
No(REF.)					
Yes	0.816	0.288	8.008	0.005	2.260(1.285–3.976)
**Constant**	-0.859	0.408	4.435	0.035	

## Discussion

This study first examined the factors related to the social network of core members of elderly care service social organizations. Our analyses revealed that gender, level of education, and awards related to elderly care services were associated with social network scores. Our data demonstrated that the median social network score of the 308 core members of social organizations in the elderly care service was 14 points, showing an urgent need for improvement of the social network level of the core members of social organizations in the field of elderly care services. Previous studies showed that core staff with richer social networks could frequently benefit from social network and had easier access to external resources and information, thus the social network could affect individual performance in organizations [24, 25]. Besides, other studies showed that proper leadership of the nursing home improves working conditions of the employees, and the leader’s social network can stimulate the creativity of the employees, which is extremely beneficial to the development of the organization [26, 27]. Moreover, another study demonstrated that core members could establish close contact with other organizations through social networks, and could acquire knowledge on work-related problems. The data showed that the leaders could easily get solutions, achieve better individual performance and that the social network of core members could determine the network of the entire organization [28]. Therefore, enriching the social network of core members of the social organization in elderly care services could enhance the development of elderly care.

Besides, the binary logistic regression analysis results showed that gender was one of the factors that affected the core members of social organizations in elderly care services, showing that the scale of men's social network was larger compared to that of women. This was partly consistent with a previous study [29], and was associated with obvious gender differentiation in social network, as well as the wider social network in men. In most cases, most leaders were male and most of the social resources and support might be more inclined to men [30, 31]. On the contrary, other studies showed that women had higher social network than men and that women’s level of informal participation in the network was higher than that of men but the level of formal participation in the network was lower than that of men [32, 33]. This phenomenon was associated with economic and cultural differences in different regions. In many countries in Eastern Europe, due to the collapse of the Soviet Union, there was a huge disappearance in social welfare. Thus, many people, especially women, turned to social network to seek social and emotional support [33].

In addition, our results revealed that core members in social organizations who had higher education or with a college degree or above were more likely to have a better social network. This was in sync with a previous study, which showed that educational level was related to social network, and those with high education levels had a higher social network [34]. Thus, well-educated people might need to spend more time in school, so had enough time to establish solid social relationships with the students and teachers, leading to enhanced social networks [35]. There is also a tendency for people to expand their network through friends with a high level of education. In addition, people could acquire more professional technical and social knowledge through education. Among the surveyed core members, only 34.1% had a college degree and above. This showed that recruitment and appointment of core members of social organizations in the elderly care services should consider individuals with higher education achievement.

Receiving awards related to elderly care services was also one of the factors associated with social network of core members of social organizations in the elderly care service organizations, and core members who had received awards had a higher social network. Receiving awards is a recognition of personal and professional ability, and could help core members gain a better reputation and popularity. There is a strong correlation between reputation and social network [36]. A good reputation could increase one's position in others, and a good personal reputation can attract the help of others and be rewarded, by both friends and strangers [37]. This kind of long-term interpersonal interaction greatly helps in the improvement of a person’s social network. Besides, people with good reputations often get more opportunities to participate in activities, thereby broadening their social network. Thus, core members of social organizations in elderly care service organizations should strengthen their management and technical learning to enhance their comprehensive capabilities.

In general, respondents who had received training related to elderly care services would have a richer social network. Our study showed that employees could acquire more knowledge through management training. Previously, it had been reported that the more the employees acquire knowledge, the faster the growth [38]. In addition, core members’ training and knowledge gain could also fuel friendships with more peers, expanding the interpersonal network in the elderly care service. Our findings did not, however, show that receiving training related to elderly care services could have an impact on the social network of core members. The longer the survey subjects worked in the elderly care service field, the more experienced and relevant they became in the provision of elderly care services. Besides, core members with a longer length of service would generally have a larger social network scale. However, the binary logistic regression analysis results did not show any differences in the core members' social network in different lengths of service groups. These findings need further exploration in follow-up studies.

This study quantitatively measured the social network level of core members of social organizations in the field of elderly care services in Anhui Province for the first time, providing a reference for future research on the social network of core members in the field of elderly care services. In the current situation of the elderly care environment, there are limited resources for the construction of elderly care services, and thus intangible resources such as social network in elderly care services could be helpful. Therefore, the use of social network is an innovative and feasible part of the social capital theory to study the participation of social organizations in elderly care services. According to the research results, we have the following suggestions. First, we should pay more attention to female core members in the field of elderly care services and strengthen the cultivation of their social network. Secondly, when recruiting core members of social organizations in the field of elderly care services, we should try to select highly educated personnel. Finally, the government and organizations should encourage and help the core members of social organizations in the field of elderly care services to apply for awards related to elderly care services, so as to enhance the reputation and popularity of core members and enrich their social network.

Whereas our study has interesting findings, it is a cross-sectional study conducted in Anhui Province, and thus the findings are only applicable to Anhui Province. In addition, we have only preliminarily explored the social network scale and related influencing factors of the core members of social organizations in the field of elderly care services in Anhui Province, and have not conducted further research on their social network level. Besides, due to time, funding, coordination, and other factors, the sample size we collected are small. More survey areas and larger sample size are needed in future studies.

## Conclusions

This study explored the factors that influence the social network of core members of elderly care service social organizations in Anhui Province, China. We demonstrated that there is a low social network among the core members of social organizations in the elderly care services in Anhui Province. Furthermore, we showed that gender, education level, and award-winning status are some of the factors that influence the social network of core members. This study provides references and suggestions for strengthening the construction of elderly care services.

## Supplementary Information

**Additional file 1.** Questionnaire for core members of social organizations in the field of elderly care services.

## Data Availability

The datasets analysed during the current study are available from the corresponding author on reasonable request.
