# Supplementary material for: Factors related to the social network of core members of elderly care service social organizations: a cross-sectional study
Source: BMC Health Serv Res. 2022 Sep 10;22:1147. doi: 10.1186/s12913-022-08545-7 (PMC9464390; doi:10.1186/s12913-022-08545-7)
Supplement: Supplementary file 1 — Additional file 1. Questionnaire for core members of social organizations in the field of elderly care services. [file 12913_2022_8545_MOESM1_ESM.docx]

**Questionnaire for core members of social organizations in the field of elderly care services**

**Part A Basic information of** **the core members**

| **A** **Basic information** |
| --- |
| **A1 Gender:** ①Male；②Female |
| **A2 Age：** |
| **A3 Nationality:** ①Han；②Others |
| **A4 Education：**①Junior high school and below ②Senior high school ③College degree and above |
| **A5 Marital status:** ①Married；②Others |
| **A6 Professional title:** ①Have；②Not have |
| **A7** **Full-time staff:** ①Yes；②No |
| **A8 Length of service in the organization:** ①≤1year; ②2-5 years; ③≥6 years |
| **A9 Length of service in the elderly care field:** ①≤1year; ②2-5 years; ③≥6 years |
| **A10 Have you obtained the practice certificate?** ①Yes；②No |
| **A11 Have you obtained a professional qualification certificate?** ①Yes；②No |
| **A12 Have you received training related to elderly care services?** ①Yes；②No |
| **A13 Have you ever been reported by the media for engaging in elderly care services?** |
| 1. Yes ②No |
| **A14 Have you received awards related to elderly care services?** ①Yes；②No |

**Part B Social network of the core members**

| **Entries:** | ①0 | ②1-5 | ③6-10 | ④11-15 | ⑤16 and above |
| --- | --- | --- | --- | --- | --- |
| **B1.1** Number of your acquaintances in the Civil Affairs Department. |  |  |  |  |  |
| **B1.2** Number of your acquaintances in the Health Committee. |  |  |  |  |  |
| **B1.3** Number of your acquaintances in the Medical Security Bureau. |  |  |  |  |  |
| **B1.4** Number of your acquaintances in other government departments. |  |  |  |  |  |
| **B1.5** Number of your acquaintances in the community committee. |  |  |  |  |  |
| **B1.6** Number of your acquaintances in the social organization federation. |  |  |  |  |  |
| **B1.7** Number of your acquaintances in other elderly care service social organizations. |  |  |  |  |  |

Investigator：

Quality controller：

Investigation time：
